# Comparison of two different nickel oxide films for electrochemical reduction of imidacloprid†

**DOI:** 10.1039/c9ra09505e

**Published:** 2020-01-16

**Authors:** Zongyu Liu, Ying Tian, Xiaohui Zhou, Xiao Liu, Liping Huang

**Affiliations:** Key Laboratory of Industrial Ecology and Environmental Engineering, Ministry of Education (MOE), School of Environmental Science and Technology, Dalian University of Technology Dalian 116024 China lipinghuang@dlut.edu.cn; Key Laboratory of Environmental Science and Technology, Education Department of Liaoning Province, College of Environmental and Chemical Engineering, Dalian Jiaotong University Dalian 116028 PR China greenhusk@126.com

## Abstract

A nickel oxide (NiO) thin film was successfully prepared on Ni foil *via* a sol–gel method and a reduced state nickel oxide (r-NiO) thin film was obtained by etching NiO with hydrazine hydrate solution. Structure characterization through X-ray diffraction and scanning electron microscopy revealed the growth of nanostructure films on the surface of nickel foil. Cyclic voltammetry, linear sweep voltammetry and electrochemical impedance spectroscopy were used to assess the performance of the two films. Electroreduction of alkaline imidacloprid solution under potentiostatic conditions was carried out in a three-electrode system. The removal efficiencies of 80.2% (r-NiO) and 66.3% (NiO), and the current efficiencies of 67.3% (r-NiO) and 58.9% (NiO) were much higher than 41.7% (removal efficiency) and 0.003% (current efficiency) on the bare Ni electrode. This study prepared two novel thin films with composites of NiO or r-NiO, and thus provided feasible and efficient electrochemical degradation of imidacloprid. The degradation products were characterized and the possible degradation pathways were proposed.

## Abstract 2

A nickel oxide (NiO) thin film was successfully prepared on Ni foil *via* a sol–gel method and a reduced state nickel oxide (r-NiO) thin film was obtained by etching NiO with hydrazine hydrate solution.

## Graphical abstract

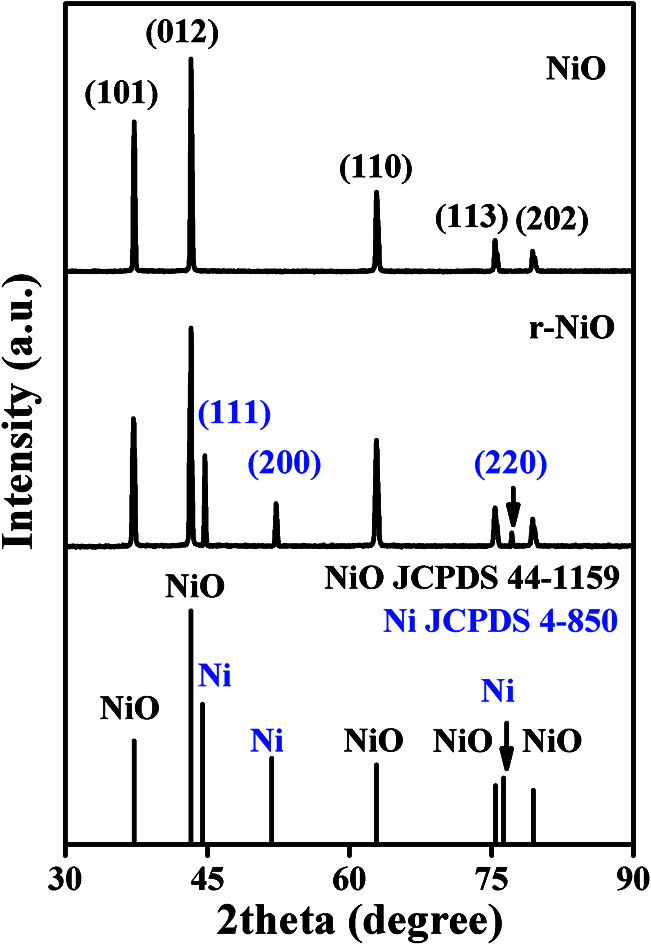

## 1. Introduction

Imidacloprid (IMD), a systemic chloronicotinyl insecticide with remarkable water solubility, high toxicity, and good stability in water, is mainly applied to agriculture for controlling sucking insects in crops such as aphids, white flies and termites, and can cause IMD pollution in water sources and soil.^1,2^

To solve the pollution problem, various treatment processes have been studied. While diverse microorganisms may potentially degrade insecticides, IMD is none biodegradable due to its high toxicity.^3,4^ A lot of work has focussed on exploring effective methods for IMD treatment. Ultraviolet (UV) photolysis with photocatalysis and photo-Fenton's reagent are the most intensively applied technologies. However, photolysis is usually not a competitive option due to the low quantum efficiency as most pesticide compounds are only partially degraded.^5,6^ Alternative to advanced oxidation processes with production of hydroxyl radicals (·OH) through hydroxyl radical attack,^7–9^ direct or indirect electrochemical generation of ·OH in electrochemical advanced oxidation processes (EAOPs) was proposed as a potential option to remove pesticides from contaminated waste water.^10–12^ This electrochemical process based on redox reactions overcomes the drawbacks of consuming extra chemical reagents or producing secondary pollution in conventional chemical or physical treatments.^13^ However, the need for expensive electrodes and thus the relatively large capital investment limits their practical application.^14^ Much effort is still needed to employ highly efficient and cost effective electrodes in order to broaden the applicable field of electrochemical degradation for IMD and other pesticide contaminated wastewater.

While electrochemical oxidation is effective for the degradation of IMD, anodes with expensive materials including TiO_2_/Ti,^15,16^ Ti/RuO_2_–TiO_2_, Ti/RuO_2_–IrO_2_–TiO_2_,^17^ Ti/SnO_2_–Sb_2_O_3_,^18^ or Pt and boron-doped diamond (BDD)^19^ were purposely used.

In these reports, electrochemical oxidation was carried out using expensive anode to resist the strong oxidative effect of hydroxyl radical. In parallel to the electrochemical oxidation, electrochemical reduction on the low-cost cathode has been scarcely reported for IMD degradation. The reasons of choosing electrochemical reduction instead of oxidation for IMD removal lies in: (i) inexpensive materials can be used as cathode electrodes due to their much less destruction by cathodic processes; (ii) cathodic reduction reactions feasibly occur with lower overvoltage, compared to anodic oxidation with higher overvoltages for ·OH generation; and (iii) species of IMD, difficultly degraded by electrochemical advanced oxidation might be feasibly removed through electrochemical reduction.

In this study, two novel materials of NiO based on Ni foils *via* a sol–gel method and r-NiO by etching NiO with hydrazine hydrate solution were prepared as cathodes and assessed for efficient IMD degradation in alkaline aqueous solution. NiO has been regarded as a promising electrode candidate due to its cost effectiveness, superior chemical and electrical stability, and transparently p-type semi-conductivity based high efficiency.^20,21^ The characterization of the oxides is investigated by X-ray diffraction (XRD), scanning electron microcopy equipped with energy dispersive spectrometer (SEM-EDS) and electrochemical tests. IMD degradation products were characterized by high performance liquid chromatograph-mass spectrometer (HPLC-MS) and the possible degradation pathways were proposed.

## 2. Experiment

### 2.1 Chemical reagents

All organic reagents were commercial products of the highest purity available (>98%). Sulfuric acid, citric acid, acetone, ethanol, *n*-butyl alcohol, sodium borohydride (NaBH_4_) and nickel dichloride hexahydrate (NiCl_2_·6H_2_O) were analytical grade. Imidacloprid (98.6%) was obtained from Agricultural Research Institute of Shanghai.

### 2.2 Preparation of NiO and r-NiO films

Specimens with dimensions of 70 × 10 × 0.1 mm cut from Ni plates were utilized as substrates. The Ni plates were sequentially and ultrasonically pretreated with acetone, hydrochloric acid (1.0 mol L^−1^ HCl), and deionized water.

The Ni plates were put at the bottom of a beaker. Then, 3.5 g of NiCl_2_·6H_2_O was dissolved in an *n*-butyl alcohol–ethanol (100 mL : 50 mL) solvent and 18.9 g of citric acid solution was added with magnetic stirring for 30 min. Afterwards, deionized water was added drop by drop. Light green sol–gel was then formed and coated on Ni plate under 90 °C heating reflux. After 60 min, the Ni plate coated sol–gel was taken out from the beaker and calcined at 300 °C for 20 min for preparation of NiO. Repeat the coating steps several times until NiO film on the surface of Ni plates were uniform. Finally, the NiO film coated on Ni plate was sequentially and ultrasonically washed with acetone and deionized water.

The as-prepared NiO film coated Ni plate was put in 10% NaBH_4_ solution for 240 min at room temperature. The r-NiO film was then obtained, and sequentially and ultrasonically washed with acetone and deionized water.

### 2.3 Electroreduction of imidacloprid

Stock solution of IMD (40 mg L^−1^) in alkaline (0.1 mol L^−1^ NaOH) was prepared with deionized water.

Electroreduction of IMD under potentiostatic condition was carried out at a series of potential (−1.0 V, −1.1 V, 1.2 V, −1.3 V and −1.4 V) in 50 mL basic solution containing 100 mg L^−1^ IMD and 0.1 mol L^−1^ NaOH for 1000 s at room temperature. The removal efficiency of imidacloprid was calculated based on eqn (1):1
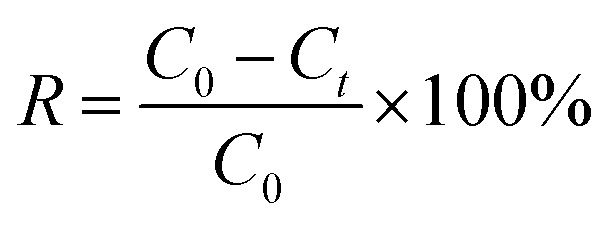
where *C*_0_ is the initial concentration of imidacloprid, *C*_*t*_ is the concentration after a certain time of electroreduction using NiO, r-NiO or Ni electrodes.

Current efficiency was calculated as eqn (2)2
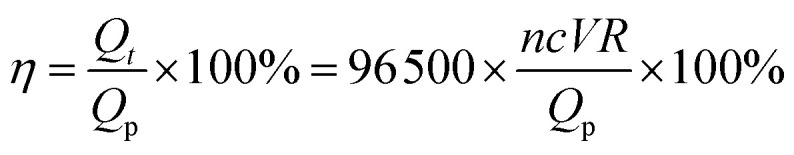
where, 96 500 is Faraday constant (C mol^−1^); *η* is the current efficiency (%); *R* is removal efficiency calculated according to eqn (1) (%); *Q*_*t*_ is the theoretical charge needed to reduce IMD (C); *Q*_p_ is the practically consumed charge for IMD reduction (C); *c* is the molar bulk concentration of imidacloprid, here is 0.000156 mol L^−1^ (100 mg L^−1^); *V* is the electrolyte volume, here is 0.05 L (50 mL); *n* is the number of electrons, here is 1.

### 2.4 Liquid chromatography analysis

The concentration of imidacloprid and its transformation products were monitored by liquid chromatography (Agilent HP 1200, Agilent Technologies, Inc., USA), equipped with an Agilent model pump, autosampler, column compartment, and an ultraviolet detector with a C-18 column with the dimensions 4.6 × 150 nm. The mobile phase used a mixture of acetonitrile and water (40 : 60) at a flow rate of 0.5 mL L^−1^. The injection volume of the sample was 5 μL, and the ultraviolet detection was *λ* = 270 nm. The samples were filtered through 0.45 μm filter paper before injection.

An HPLC-MS (Agilent 1100-6224) system was used to analyze the transformation products obtained in imidacloprid degradation, with an LC column Luna 5C18100A. The samples were filtered through 0.45 μm filter paper before injection. In this case, the isocratic eluent was 98% (1 nmol L^−1^ aqueous sodium formate and 0.1% formic acid) and 2% acetonitrile (0.1% of formic acid), which was pumped at a rate of 0.4 mL min^−1^ for 80 min. Detection was carried out with the diode array detector set at 270 nm and the column temperature maintained at 35 °C.

### 2.5 Structure characterization and electrochemical tests

The as-prepared NiO and r-NiO were characterized through XRD performed on a Panalytical Empyrean diffractometer with Cu Kα radiation. The morphology and the elements of the surface of NiO and r-NiO were analyzed by SEM-EDS (JSM-6360LV).

For electrochemical tests, a three-electrode system was employed. NiO, r-NiO film coated Ni plates or the bare Ni plate were used as working electrodes, whereas a platinum plate and a saturated calomel electrode (SCE) were used as counter and reference electrodes, respectively in electrolyte of NaOH aqueous solution (0.1 mol L^−1^). Cyclic voltammetry (CV) was performed at a scan rate of 50 mV s^−1^ between −0.4 V and 0.3 V. Linear sweep voltammetry (LSV) was performed at −1.4 V to 0 V at a scan rate of 5 mV s^−1^. The electrochemical impedance spectroscopy (EIS) test was carried out in a frequency range of 0.01 Hz to 100 kHz. All the electrochemical measurements were performed on an electrochemical work station (PARSTAT2273, Princeton Applied Research, USA) at 20 °C.

## 3. Results and discussion

### 3.1 Structural characterization of NiO and r-NiO films

**Fig. 1:**
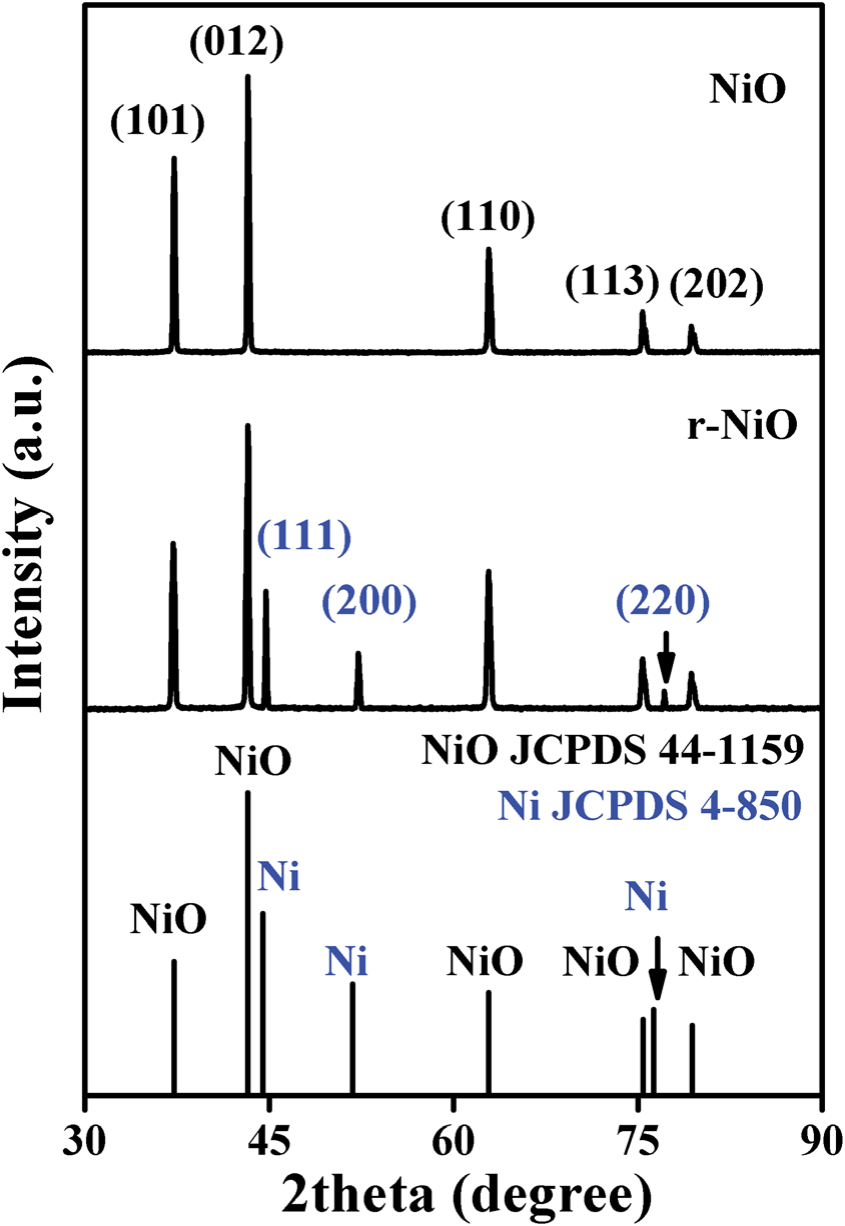
XRD patterns of NiO and r-NiO films coated on Ni plate.

The structure characterizations of the NiO and r-NiO films were analyzed by XRD (Fig. 1). The peaks at 2*θ* angle values of 37.2°, 43.2°, 62.8°, 75.4° and 79.3° corresponded to characteristics of nickel oxide, which could be assigned to (101), (012), (110), (113) and (202) reflection of NiO (JCPDS 44-1159), respectively. The peaks of Ni foils were not observed in the diffraction pattern of NiO. For the diffraction pattern of r-NiO, however, the peaks at 2*θ* angle values in the same positions of NiO were decreased slightly. Meanwhile, three remarkable peaks at 44.5°, 51.8° and 76.3° corresponded to characteristics of nickel, which could be assigned to (111), (200) and (220) reflection of Ni (JCPDS 4-850) respectively. These results collectively demonstrated the difference of r-NiO and NiO films each other.

The grain sizes of NiO and r-NiO were calculated using Scherrer equation:3
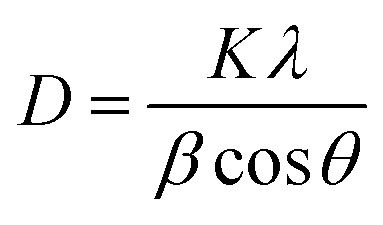
where *D* is crystallite size, *λ* is the wavelength of X-ray radiation, *β* is the full width at half height of symmetrical shape of the diffraction peak and *θ* is the Bragg angle. The average grain size of NiO film was about 50 nm, while r-NiO film was about 30 nm, suggesting r-NiO finer nanocrystalline film than NiO by reduction of NaBH_4_ solution.

**Fig. 2:**
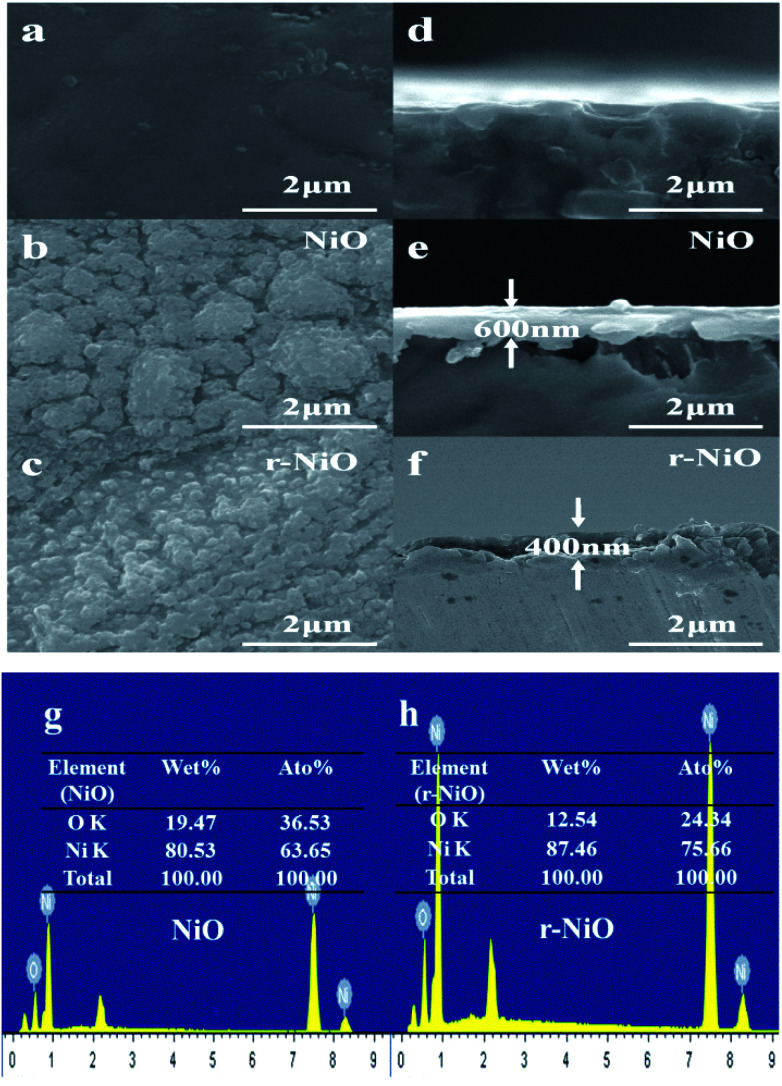
SEM images of surface view (a–c) and tilted view (d–f) of Ni, NiO and r-NiO, EDS spectra of NiO (g) and r-NiO (h).

Fig. 2 showed the SEM images of the surface views and the corresponding tilted views of Ni substrate, and NiO and r-NiO films. Both NiO and r-NiO films were composed of granular particles with the size of about 0.2–2 μm. However, r-NiO film exhibited thinner thickness and smaller particles than NiO film, meaning better conductivity for the former.^22^ The morphology of even r-NiO thin films demonstrated that the etching by NaBH_4_ played an important role in the formation of r-NiO nanocrystals.

EDS analysis showed that the NiO thin films consisted of 80.5% (w) of Ni element and 19.5% (w) of O element. However, r-NiO film consisted of higher Ni elements of 87.5% (w) and lower O element of 12.5% (w) due to the reduction effect of NaBH_4_. This result was well consistent with the result in the XRD patterns (Fig. 1), indicating that r-NiO can effectively improve the structure characteristics and thus the enhanced the electrochemical performance demonstrated hereinafter.

### 3.2 Electrochemical performance of NiO and r-NiO electrodes

**Fig. 3:**
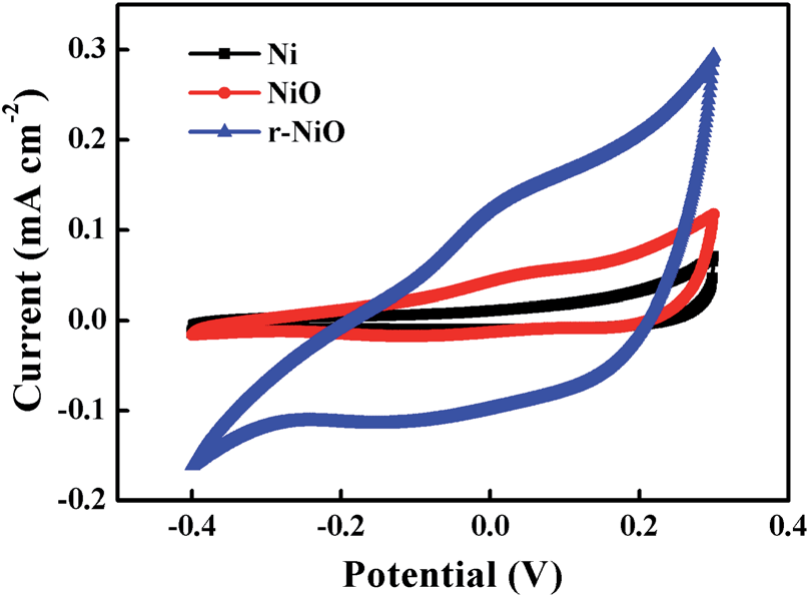
CV curves obtained on NiO, r- NiO and Ni electrodes from −0.4 to 0.3 V at a scan rate of 50 mV s^−1^ in 0.1 mol L^−1^ NaOH solution.

The electrochemical tests of CV, LSV and EIS were carried out to investigate the characteristics of NiO and r-NiO films. Fig. 3 presented the CV curves of different electrodes in the range of −0.4 V to 0.3 V. Both the r-NiO and the NiO films showed much larger and more symmetric current response than the Ni electrode, suggesting the faster electron-transfer kinetics and the better electrochemical catalytic activity than the bare electrode by the formation of the crystallization films. In addition, the r-NiO film displayed much larger current values than the NiO film, mainly ascribed to the former thinner film and favorable morphology (Fig. 1 and 2).

**Fig. 4:**
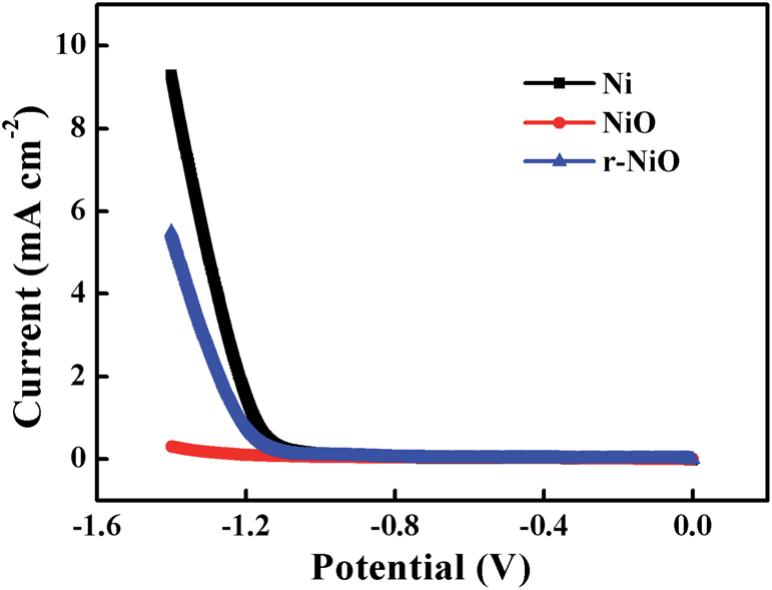
LSV curves obtained on Ni, NiO and r-NiO electrodes from −1.4 to 0 V at a scan rate of 5 mV s^−1^ in 0.1 mol L^−1^ NaOH solution.

LSV curves demonstrated that Ni electrode showed a larger current sharp than NiO or r-NiO at potentials more negative than −1.15 V (Fig. 4), mainly attributed to strong hydrogen evolution in this region. These results precisely demonstrated that hydrogen evolution was greatly reduced by the NiO or r-NiO films, compared to the bare Ni. This merit of the NiO or r-NiO films favored for the electroreduction of imidacloprid and improved the current efficiency because of the alleviation of the side reaction of hydrogen evolution at the negative potentials.^23^ Moreover, the crystallization of the NiO and r-NiO films exhibited much better electrochemical catalytic activity for imidacloprid reduction than the bare Ni demonstrated hereinafter. The r-NiO film exhibited a larger current response than NiO film, presumably due to its thinner thickness, higher conductivity and better electrochemical activity than the former.

**Fig. 5:**
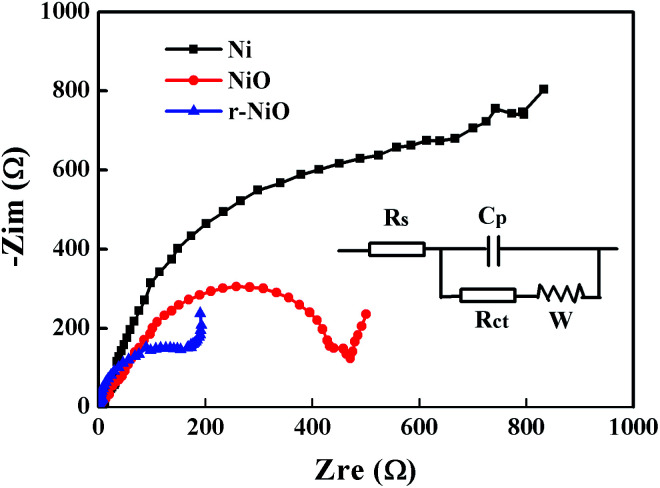
Nyquist plots of Ni, NiO, r-NiO electrodes in 0.1 mol L^−1^ NaOH solution (inset: fitting equivalent circuit).

The Nyquist plots of EIS spectra for NiO and r-NiO films (Fig. 5) were characteristic of semicircle, which was significantly different from the bare Ni, suggesting the growth of nanostructure films on the surface of nickel foil. An intercept at high frequency region with real axis of *Z*′ is inner resistance (*R*_s_) and distorted semicircle is charge-transfer resistance (*R*_ct_) across the electrode–electrolyte interface.^24,25^ From the fitting equivalent circuit (inset of Fig. 5), it can be concluded that the *R*_s_ of NiO thin film electrode is about 0.89 Ω, while the *R*_s_ of r-NiO is about 0.85 Ω. The span of the semicircle along the *x*-axis from high to low frequency represented the *R*_ct_. The smaller the diameter of the semicircle, the better the electrochemical activity. The r-NiO shows much smaller *R*_ct_ of 220 Ω cm^2^ than 465 Ω cm^2^ for NiO. The better conductivity and less *R*_ct_ of the r-NiO film can be ascribed to its thinner thickness and smaller particles. As a consequence, the r-NiO thin film exhibited better electrochemical activity and higher electrochemical reduction ability than the NiO film, as subsequently demonstrated.

### 3.3 Electroreduction of imidacloprid

**Fig. 6:**
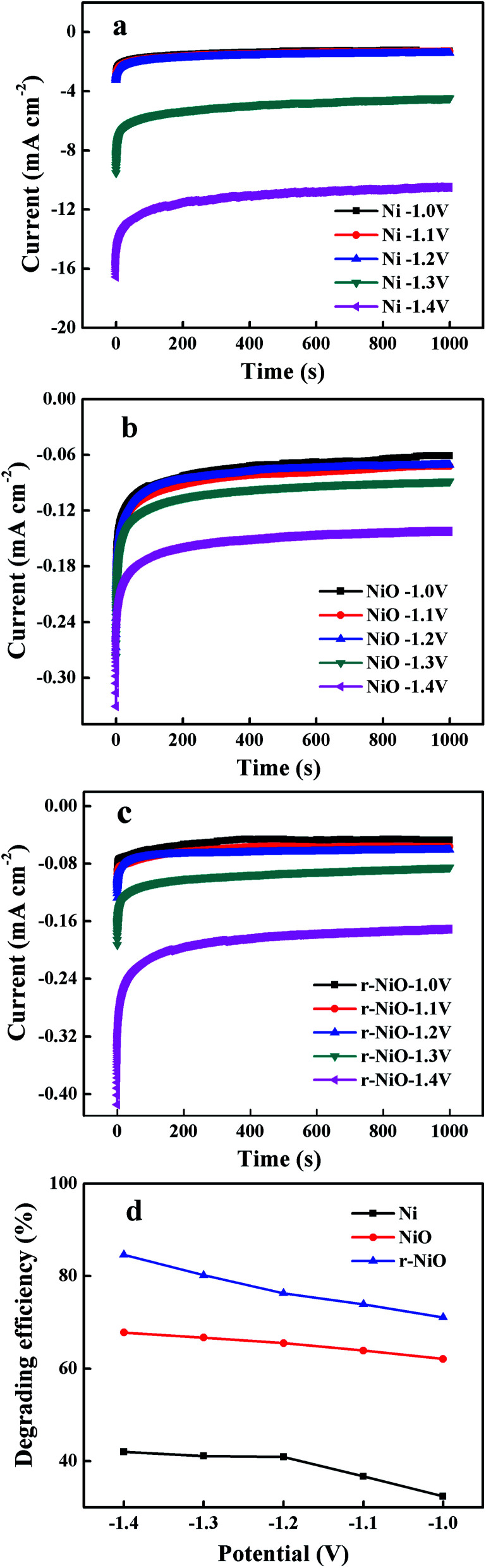
Current *vs.* time curves obtained on electrodes of (a) Ni, (b) NiO, (c) r-NiO and (d) degradation efficiency of imidacloprid of the three electrodes at different potentials in 0.1 mol L^−1^ NaOH solution.

For all electrodes of Ni, NiO and r-NiO, degradation efficiency of IMD increased with the more negative potential (Fig. 6d), due to the abundant electrons supply at more negative potentials. At the highly negative potentials, hydrogen was observed to evolve drastically on the bare Ni electrode (Fig. 6a), consistent with the much larger current response obtained on Ni electrode (Fig. 6b and c), while hydrogen evolution were slightly generated on NiO and r-NiO electrodes. This result implied the undesired side reaction of hydrogen evolution has been greatly reduced on the NiO or r-NiO films.^26^ In addition, better electrochemical catalytic activity could be achieved by the formation of crystallization films of NiO or r-NiO. As a result, the removal efficiencies of 80.2% (r-NiO) and 66.3% (NiO), and the current efficiencies of 67.2% (r-NiO) and 58.9% (NiO) were much higher than 41.7% and 0.003% on the bare Ni electrode at −1.3 V (Fig. 6d and Table 1). The values obtained on r-NiO were higher than on NiO, explaining the better electrochemical catalytic activity and the lower charge transfer resistance in the former.

**Table 1:** Comparison of degradation efficiency and current efficiency by potentiostatic reduction at −1.3 V on different electrodes

Electrode	Removal efficiency/%	Current efficiency/%
Ni	41.7	0.003
NiO	66.3	58.9
r-NiO	80.2	67.2

Comparing these results with literature, the removal rate of IMD (equivalent to 4.81 mg L^−1^ min^−1^) observed in this study at an initial IMD of 100 mg L^−1^ and much lower current density of 0.10 mA cm^−2^, was much higher than the rate achieved on a boron-doped diamond anode at a much higher current density by EAOPs.^27–29^ In addition, ·OH generation on anodic oxidation during EAOPs processes at high-voltage anodes means high electric price or low current efficiency compared to the results of this study with fairly high current efficiency.

The main possible reactions involved in the electrochemical reduction processes were as follows:^30^

4 2H_2_O + 2e → H_2_↑ + OH^−^

5 Imidacloprid + e → reduction products

6 Imidacloprid + [H] → reduction products

In alkaline solution, the IMD was reduced by direct reduction with accepting electrons (eqn (5)) or by indirect reduction by reaction with the freshly generated atom hydrogen ([H]) (eqn (6)) generated on the surface of the electrode, and then turned into transitional products and finally terminal products.

In alkaline solution, the IMD was reduced by direct reduction with accepting electrons (eqn (5)) or by indirect reduction by reaction with the freshly generated atom hydrogen ([H]) (eqn (6)) generated on the surface of the electrode, and then turned into transitional products and finally terminal products. The occurrence of the side reaction of more hydrogen evolution through water hydrolysis (eqn (4)) on the surface of the Ni electrode than that on the NiO or r-NiO films, explained the higher current efficiencies in the latter (Table 1).

**Fig. 7:**
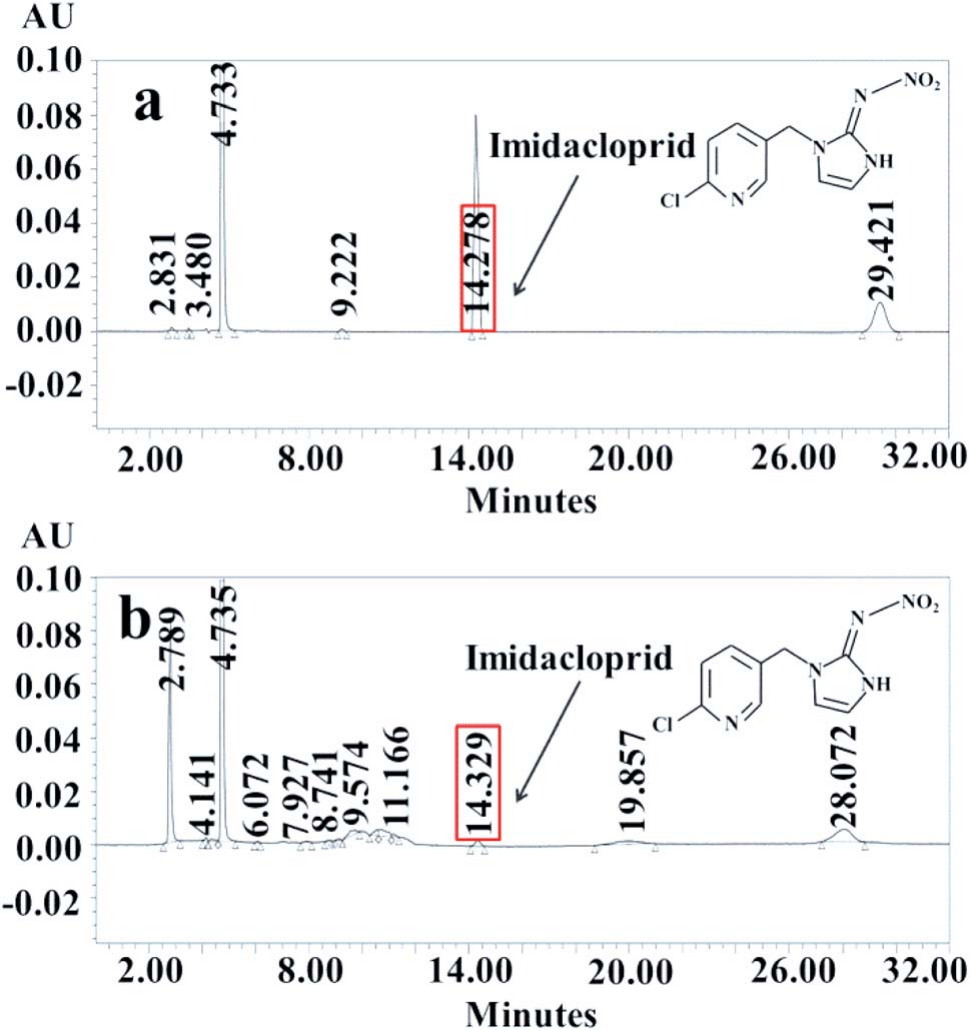
Analysis results of HLLC-MS before (a) and after (b) electroreduction of imidacloprid at −1.3 V in 0.1 mol L^−1^ NaOH for 1000 s.

Fig. 7 showed the intermediates identified by HPLC-MS after electroreduction of IMD with r-NiO electrode. Apparent decrease in peak height of IMD along with the appearance of some new peaks was observed, demonstrating the decrease of IMD and the appearance of new intermediates by electrochemical degradation.

**Fig. 8:**
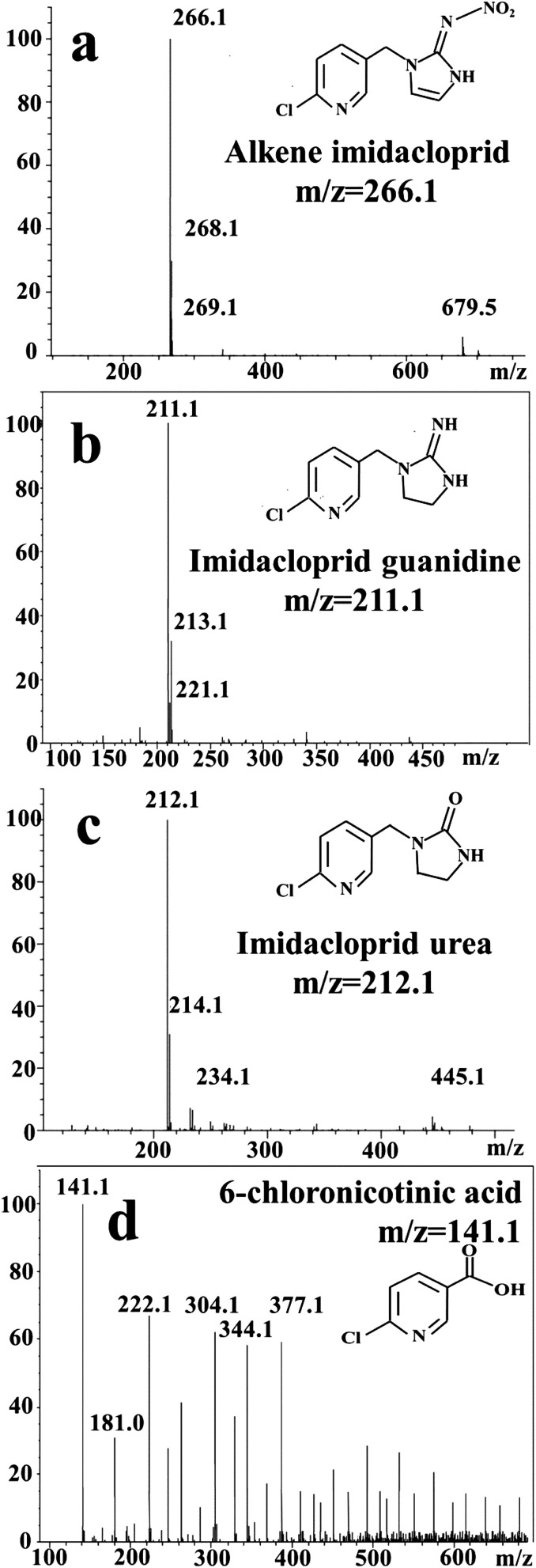
MS of intermediates after electroreduction of imidacloprid by r-NiO electrode.

The intermediates identified by mass spectra (MS) indicated IMD was degraded to alkene IMD (*m*/*z* = 266.1), imidacloprid guanidine (*m*/*z* = 211), IMD urea (*m*/*z* = 212) and 6-chloronicotinic acid (*m*/*z* = 141) (Fig. 8).

**Fig. 9:**
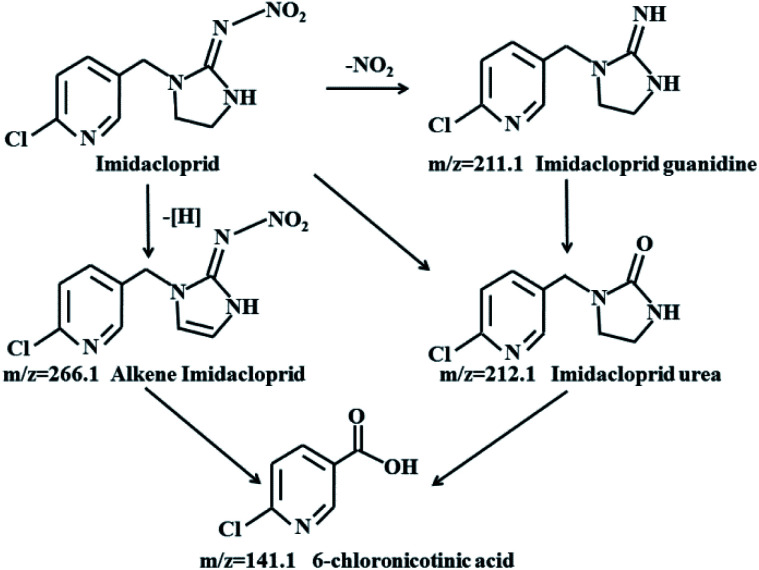
The proposed pathway for electroreduction of imidacloprid by r-NiO electrode.

Based on the detected intermediates, a pathway for electrochemical degradation of IMD with r-NiO electrode was proposed (Fig. 9). The charge transfer led to the formation of alkene IMD upon elimination of H atoms.^31^ The alkenes were further degraded to yield the 6-chloronicotinic acid, which exhibited a higher insecticidal activity than IMD. However, this 6-chloronicotinic acid displayed a large degree of mineralization and lost its acute toxicity with extended time.^32^ IMD urea was the principal degradation product by hydrolysis and in weak alkaline media, which has been proven to have lower toxicity than the parent compound and can be easily mineralized.^33,34^ IMD guanidine was formed by the loss of the –NO_2_ group after H^+^ attacking the moiety of N–NO_2_ of IMD,^35^ which showed higher mammalian toxicity than the parent compound. However, this intermediate could eventually be converted into nontoxic molecular fragments in the natural environment. The 6-chloronicotinic acid was the major degradation product of IMD with simple chemical structure, and had less ecotoxicity than the IMD.^36^ More importantly, this compound can be easily mineralized into CO_2_ and H_2_O under acid conditions.

## 4. Conclusions

The novel films of NiO prepared by sol–gel method and the r-NiO by reduction of NiO film were successfully explored for electrochemical reduction of IMD. While NiO and r-NiO crystallization films were composed of granular particles, the r-NiO film exhibited thinner thickness, smaller particles and better conductivity than the NiO film. The NiO and r-NiO films also displayed the merit of the alleviation of the side reaction of hydrogen evolution at the negative potentials. Electroreduction of alkaline IMD solution under potentiostatic conditions was successfully carried out in a three-electrode system, achieving removal efficiencies of 80.2% (r-NiO) and 66.3% (NiO), current efficiencies of 67.3% (r-NiO) and 58.9% (NiO), much higher than 41.7% (removal efficiency) and 0.003% (current efficiency) on the bare Ni electrode. The higher removal efficiencies were mainly ascribed to the better electrochemical catalytic activity and the lower charge transfer resistance of the r-NiO and the NiO films, whereas the higher current efficiencies were due to the alleviation of the side reactions of hydrogen evolution during the IMD removal process. Four degradation intermediates were detected and the possible pathway for electrochemical degradation of IMD was proposed. The novel nickel oxide prepared on Ni foil was proved to be feasible and cost effective for efficient electrochemical degradation of IMD.

## Conflicts of interest

There are no conflicts to declare.

## Supplementary Material

RA-010-C9RA09505E-s001

RA-010-C9RA09505E-s002

RA-010-C9RA09505E-s003

RA-010-C9RA09505E-s004

RA-010-C9RA09505E-s005

RA-010-C9RA09505E-s006

RA-010-C9RA09505E-s007

RA-010-C9RA09505E-s008

RA-010-C9RA09505E-s009
