# Supplementary material for: Comparison of two different nickel oxide films for electrochemical reduction of imidacloprid
Source: RSC Adv. 2020 Jan 16;10(6):3040–7. doi: 10.1039/c9ra09505e (PMC9049129; doi:10.1039/c9ra09505e)
Supplement: RA-010-C9RA09505E-s005 [file RA-010-C9RA09505E-s005.rtf]

Name and formula

Reference code:	00-044-1159 

Compound name:	Nickel Oxide 

Empirical formula:	NiO
Chemical formula:	NiO


Crystallographic parameters

Crystal system:	Rhombohedral 
Space group:	R-3m 
Space group number:	166

a (?):	  2.9552 
b (?):	  2.9552 
c (?):	  7.2275 
Alpha (¡ã):	 90.0000 
Beta (¡ã):	 90.0000 
Gamma (¡ã):	120.0000 

Calculated density (g/cm^3):	  6.81 
Volume of cell (10^6 pm^3):	 54.66 
Z:	  3.00 

RIR:	  2.10 


Subfiles and quality

Subfiles:	Alloy, metal or intermetalic
	Corrosion
	Forensic
	Inorganic
Quality:	Star (S)

Comments

Color:	Light green  
Creation Date:	1970/1/1 
Modification Date:	1970/1/1 
Sample Preparation:	Sample obtained from Merck (puriss.).  For better crystallization, annealed at 1300 C for 5 hours and quenched 
Color:	Light green 
Temperature of Data Collection:	Pattern taken at 22 C 
Additional Patterns:	Second cubic polymorph known (see 00-004-0835) 
Additional Patterns:	To replace 00-022-1189. 

 

References

Primary reference:	Wies, S., Eysel W., Mineral.-Petrograph., Institut der Universitaet Heidelberg, Germany., ICDD Grant-in-Aid, (1992)

Peak list

No.    h    k    l      d [A]     2Theta[deg] I [%]   
  1    1    0    1      2.41197    37.249      60.0
  2    0    1    2      2.08849    43.287     100.0
  3    1    1    0      1.47733    62.854      30.0
  4    1    0    4      1.47607    62.914      25.0
  5    1    1    3      1.25955    75.406      14.0
  6    2    0    2      1.20624    79.374       9.0
  7    0    0    6      1.20460    79.504       4.0
  8    0    2    4      1.04433    95.055       6.0
  9    2    1    1      0.95882   106.909       4.0
 10    2    0    5      0.95817   107.013       2.0
 11    1    0    7      0.95761   107.104       2.0
 12    1    2    2      0.93437   111.057       7.0
 13    1    1    6      0.93363   111.188       7.0
 14    2    1    4      0.85282   129.174       7.0
 15    0    1    8      0.85187   129.443       3.0
   
   
Stick Pattern

                                                             
                                                             
