# Supplementary material for: Comparison of two different nickel oxide films for electrochemical reduction of imidacloprid
Source: RSC Adv. 2020 Jan 16;10(6):3040–7. doi: 10.1039/c9ra09505e (PMC9049129; doi:10.1039/c9ra09505e)
Supplement: RA-010-C9RA09505E-s006 [file RA-010-C9RA09505E-s006.rtf]

Name and formula

Reference code:	00-004-0850 

Mineral name:	Nickel, syn 
Compound name:	Nickel 

Empirical formula:	Ni
Chemical formula:	Ni


Crystallographic parameters

Crystal system:	Cubic 
Space group:	Fm-3m 
Space group number:	225

a (?):	  3.5238 
b (?):	  3.5238 
c (?):	  3.5238 
Alpha (¡ã):	 90.0000 
Beta (¡ã):	 90.0000 
Gamma (¡ã):	 90.0000 

Calculated density (g/cm^3):	  8.91 
Volume of cell (10^6 pm^3):	 43.76 
Z:	  4.00 

RIR:	-


Subfiles and quality

Subfiles:	Alloy, metal or intermetalic
	Common Phase
	Educational pattern
	Forensic
	Inorganic
	Mineral
	NBS pattern
Quality:	Star (S)

Comments

Color:	White  
Creation Date:	1970/1/1 
Modification Date:	1970/1/1 
Color:	White 
Sample Source or Locality:	Sample obtained from Johnson Matthey Company, Ltd 
Analysis:	Spectrographic analysis show <0.01% each of Mg, Si and Ca 
Temperature of Data Collection:	Pattern taken at 26 C 
Additional Patterns:	See ICSD 64989 (PDF 01-087-0712). 

 

References

Primary reference:	Swanson, Tatge., Natl. Bur. Stand. (U.S.), Circ. 539, I, 13, (1953)

Peak list

No.    h    k    l      d [A]     2Theta[deg] I [%]   
  1    1    1    1      2.03400    44.508     100.0
  2    2    0    0      1.76200    51.847      42.0
  3    2    2    0      1.24600    76.372      21.0
  4    3    1    1      1.06240    92.947      20.0
  5    2    2    2      1.01720    98.449       7.0
  6    4    0    0      0.88100   121.936       4.0
  7    3    3    1      0.80840   144.679      14.0
  8    4    2    0      0.78800   155.666      15.0
   
   
Stick Pattern

                                                             
                                                             
